# The epidemiology of low back pain in chiropractors and chiropractic students: a systematic review of the literature

**DOI:** 10.1186/s12998-024-00559-8

**Published:** 2024-11-26

**Authors:** Lauren Ead, Jessica Wong, Sheilah Hogg-Johnson, Silvano Mior, Joshua Plener, Pierre Côté

**Affiliations:** 1 Institute for Disability and Rehabilitation Research, Faculty of Health Sciences, Ontario Tech University, 200 Simcoe St N, Oshawa, ON L1G 0C5 Canada; 2 Graduate Studies, Canadian Memorial Chiropractic College, 6100 Leslie Street, Toronto, ON M2H 3J1 Canada; 3 Institute for Health Policy, Management and Evaluation, University of Toronto, 155 College St 4th Floor, Toronto, ON M5T 3M6 Canada; 4 School of Physical Therapy, Faculty of Health Sciences, Western University, 1201 Western Rd, London, ON N6G 1H1 Canada; 5 Department of Research and Innovation, Canadian Memorial Chiropractic College, 6100 Leslie Street, Toronto, ON M2H 3J1 Canada; 6 Department of Clinical Education, Canadian Memorial Chiropractic College, 6100 Leslie Street, Toronto, ON M2H 3J1 Canada

**Keywords:** Chiropractors, Chiropractic students, Low back pain, Epidemiology, Prevalence

## Abstract

**Background:**

Chiropractors and chiropractic students commonly report low back pain (LBP). However, the burden of LBP in this occupational group has not been synthesized in the literature. This systematic review aims to describe the epidemiology of LBP in chiropractors and chiropractic students.

**Methods:**

We searched MEDLINE, Embase, CINAHL, and PsycINFO from inception to May 1, 2023. Eligible studies were cross-sectional, cohort, or case–control studies investigating the prevalence, incidence, associated factors, or risk factors of LBP in chiropractors or chiropractic students. Reviewers independently screened articles and assessed risk of bias using the appropriate JBI Checklists for the observational study design. We descriptively synthesized studies that were rated as low or moderate risk of bias.

**Results:**

Of 2012 citations screened, we included 2 cross-sectional studies in the evidence synthesis (1 study rated as moderate risk of bias on chiropractors, and 1 rated as low risk of bias on chiropractic students). For chiropractors, the 12-month prevalence of work-related overuse injuries to the low back was 35.6% (95% CI 29.1, 42.0) in women and 22.4% (95% CI 16.3, 29.6) in men. The 12-month prevalence of work-related acute physical injuries to the low back in chiropractors were 3.4% (95% CI 1.6, 6.8) for women and 0.7% (95% CI 0.1, 3.7) for men. Among chiropractic students, the 1-week prevalence of LBP was 69% (95% CI 64.8, 73.0). This was higher among female students (72.5%, 95% CI 67.1, 77.4) and lower among male students (64%, 95% CI 57.0, 70.6).

**Conclusion:**

There is limited high-quality evidence on the epidemiology of LBP in chiropractors and chiropractic students. Our systematic review provides a synthesis of the body of literature, highlighting that chiropractors and chiropractic students commonly report LBP. Future high-quality research is needed to address the incidence, associated factors, and risk factors of LBP.

**Supplementary Information:**

The online version contains supplementary material available at 10.1186/s12998-024-00559-8.

## Background

LBP is one of the most common work-related injuries, with a complex etiology involving biological, psychological, and social factors [1]. Globally, LBP arising from occupational ergonomic factors accounted for 24% of disability-adjusted life years in 2019 [2]. Beyond ergonomic factors, sociodemographic characteristics, and modifiable lifestyle behaviours, such as smoking, significantly contribute to the prevalence of LBP globally [2]. Understanding the complexity of contributing factors to LBP among occupations is essential because LBP is the leading cause of lost workdays among musculoskeletal conditions [1, 3]. LBP can negatively impact the physical, psychological, and social well-being of workers, contributing to disability [1].

Work-related LBP is a significant problem in healthcare providers, and many healthcare professionals such as nurses, dentists, and physiotherapists report work-related LBP [4–8]. A systematic review of allied health professionals found that the low back was the most commonly reported work-related location of injury, with prevalence estimates ranging from 6.6% to 83% [5]. Globally, the prevalence of LBP in physiotherapists was 40.1% (95% CI: 32.2 – 48.0) [9]. Historically, the prevalence of LBP in chiropractors has received little attention, but limited research suggests that chiropractors commonly report work-related LBP [10–13].

Chiropractic, a healthcare discipline taught in a number of institutions worldwide, is recognized in 90 of the 193 member nations of the United Nations [14]. Chiropractors are healthcare providers who diagnose and manage individuals with musculoskeletal disorders [15], and they are regularly exposed to physical and psychosocial stressors that could contribute to the etiology of LBP. Mior & Diakow (1986) were the first to report LBP among chiropractors in Canada with a prevalence of 74%; however, the timeframe of their prevalence is unknown [12]. Similar findings have been observed in more recent studies, with chiropractors commonly reporting work-related low back injuries [10, 11, 13]. However, results from existing studies need to be interpreted with caution because of potential sources of bias [10–13]. Additionally, emerging evidence suggests that LBP begins in their training [16–18]. This is significant because experiencing LBP as a student could negatively impact their future career and predispose them to future episodes of LBP [19]. Therefore, understanding the course of LBP throughout a chiropractor’s career, is essential to inform the development of future research on strategies to mitigate LBP.

To date, no systematic review has synthesized the burden of LBP and associated risk factors in chiropractic students and chiropractors. Understanding this burden is an important preliminary step to inform the future study of the etiology of LBP in this occupational group.

## Objectives

The objectives of our systematic review were to determine: 1) the prevalence and associated factors of LBP among chiropractors and chiropractic students, and 2) the incidence and risk factors of LBP among chiropractors and chiropractic students.

## Methods

### Study design and registration

We registered our protocol with Open Science Framework (https://osf.io/b9q2d) and reported our systematic review using the Preferred Reporting Items for Systematic Review and Meta-Analyses (PRISMA) statement checklist (see Additional File 1) [20]. We chose a systematic review design over a scoping review because our aim was to derive valid information on the epidemiology of low back pain in chiropractors and chiropractic students by critically appraising the literature and synthesizing the best available evidence.

## Eligibility criteria

Eligible studies met the following inclusion criteria: 1) published from database inception to May 1st, 2023, in a peer-reviewed journal; 2) English, French, Chinese, or Italian language; 3) cohort study, case–control study, cross-sectional study, or quantitative component of a mixed methods study if it is a cohort, case–control, or cross-sectional design; 4) investigated chiropractors or chiropractic students; and 5) investigated risk factors or factors associated with LBP or the prevalence or incidence of LBP. We excluded randomized controlled trials, conference proceedings, editorials, letters to editors, books, guidelines, lectures and addresses, meeting abstracts, consensus development statements, case reports, case series, systematic reviews, narrative reviews, laboratory studies, qualitative studies, studies not reporting methodology, and cadaveric or animal studies.

### Population

Our target population included chiropractors and chiropractic students. A chiropractor is an individual licensed in chiropractic, while a chiropractic student is enrolled in an institution that teaches chiropractic. Chiropractic is “a health profession concerned with the diagnosis, treatment, and prevention of mechanical disorders of the musculoskeletal system” [15].

### Risk factors and associated factors

We included studies that investigated risk factors or factors associated with LBP. Risk factors or associated factors included but were not limited to health-related factors, lifestyle behaviours, sociodemographic factors, and occupational factors [1, 10, 11, 21–24].

### Outcome—low back pain

We included studies that investigated the prevalence or incidence (e.g., onset of the first or new episode during a specified time interval) of LBP with or without radiculopathy. The low back is the area between the lower costal margin and the inferior gluteal fold [25]. Non-specific LBP is pain with no known pathoanatomical cause, whereas LBP with radiculopathy is characterized by leg pain along a specific lumbar nerve root with reflex loss, motor weakness, or sensory loss [1]. Studies that labelled non-specific LBP as mechanical LBP, lumbago, lumbar radiculopathy, lumbar disc herniation, sacroiliac joint syndrome, sciatica, or lumbar, lumbosacral or lumbopelvic sprain or strain were included. We excluded studies that included individuals with LBP due to pathologies such as infection, traumatic injury such as fractures, inflammatory arthropathy, or neoplasm [1]. Moreover, studies that investigated multiple musculoskeletal conditions were eligible if their results were stratified for LBP.

## Data sources and search strategies

We searched MEDLINE, CINAHL, PsycINFO, and Embase from database inception until May 1, 2023. The search strategy was developed with an experienced health sciences librarian and peer reviewed by a second librarian using the Peer Review of Electronic Search Strategies Checklist [26]. The search strategy included key words and subject headings related to 1) chiropractors and chiropractic students, 2) risk factors or associated factors, 3) prevalence or incidence, 4) low back pain, and 5) study design (see Additional File 2). There were no limits on language for the search as a list of possibly relevant articles published in excluded languages would be included in the appendix. The search strategy was adapted for each database. We used EndNote to organize citations identified from the databases and to remove and record the number of duplicates.

## Study selection

Two reviewers independently screened the title and abstracts and categorized studies as possibly relevant or irrelevant based on the eligibility criteria. We trained reviewers for screening using a random sample of 50 titles/abstracts and 25 full-text articles for eligibility. Reviewers had to achieve agreement ≥ 80% before beginning screening. Reviewers met to discuss disagreements and reach consensus, and a third reviewer was involved if consensus could not be reached. Studies deemed possibly relevant were included for full-text screening during which they were categorized as relevant or irrelevant. Reviewers documented the reason for excluding a study. If consensus was not reached, a third reviewer was consulted to decide on eligibility.

## Methodological quality and risk of bias assessment

We used the JBI (formerly the Joanna Briggs Institute) Checklist for Analytical Cross Sectional Studies [27] and the JBI Checklist for Prevalence Studies [28] to critically appraise eligible studies. Both checklists were used to separately assess any potential bias in analytic cross-sectional studies that reported both prevalence and associated factors. We used the JBI Checklist for Cohort Studies and the JBI Checklist for Case Control Studies [27]. Two reviewers independently appraised a random sample of five articles as training using JBI Checklist [27, 28]. Reviewers had to achieve agreement ≥ 80% on the overall quality based on independent appraisals before beginning the risk of bias assessment. The trained reviewers independently critically appraised eligible studies and assessed the presence of confounding, selection bias, measurement bias, and bias related to statistical analysis.

Reviewers used these instruments to inform their judgement on the internal validity of eligible studies and classified each study as low, moderate, or high risk of bias. No specific quantitative thresholds were applied to assess the overall risk of bias. Instead, the overall risk of bias was determined by evaluating each item on the tool. A study was considered to have low risk of bias if reviewers judged that potential selection bias, measurement bias, and confounding were minimal or acceptable. Particularly, reviewers emphasized potential biases related to the participant recruitment, response rate, coverage bias, and identification and measurement of the condition (e.g., whether a standard definition for LBP was used). Studies rated as low or moderate risk were categorized as ‘include’ on the JBI Checklists, while those rated as high risk of bias were categorized as ‘exclude’. Reviewers met to discuss disagreements and reach consensus, and a third reviewer was involved if consensus could not be reached.

## Data items and data collection

Two reviewers independently extracted data from five studies as a training exercise and met to resolve any disagreements. We tested the quality of data extraction on a random sample of five citations. Reviewers had to achieve agreement ≥ 80% on each item of the form before commencing data extraction. The lead author extracted the data from the remaining studies. A second reviewer verified all data extraction items by checking the accuracy of extracted data. Reviewers extracted the following data: authors, year of publication, country, location/setting, study design, target population, sample size, years in practice, years since graduation, patient contact hours per week, year of study, age, sex, gender, language spoken, recruitment method, response rate, definition of low back pain, measurement of the condition, definition of risk factors and associated factors, effect estimates (e.g., prevalence ratios, odds ratios, relative risks [crude and adjusted]), and the prevalence and incidence of LBP and its associated 95% confidence interval (CI).

## Analysis and synthesis of evidence

We computed the inter-rater agreement for screening and risk of bias assessments using the percent agreement and Cohen’s Kappa statistic. We descriptively synthesized results of studies following the guidance from Synthesis Without Meta-Analysis (SWiM) for systematic reviews checklist [29]. We assessed clinical and methodological heterogeneity among studies. Clinical heterogeneity was assessed by stratifying the results by chiropractors and chiropractic students. We assessed methodological heterogeneity across studies by categorizing studies as low or moderate risk of bias, or high risk of bias, based on the overall judgement from the JBI Checklists. Since post-hoc sample size calculations can be misleading, we considered the related JBI Checklist question less relevant for risk of bias assessment and instead used CIs to evaluate data precision [30]. We synthesized included studies rated as low to moderate risk of bias, as studies with high risk of bias are more likely to yield biased estimates. We reported the prevalence or incidence of LBP as percentages with their 95% CI. If the CI was not reported, we calculated the CI for proportions using the Wilson score method without continuity correction [31]. Effect estimates (crude and adjusted) were reported as odds ratios, prevalence ratios, or relative risks. We did not conduct a meta-analysis due to the heterogeneity of studies.

## Protocol deviation

We originally indicated that two reviewers would independently extract the data for all eligible studies. However, after our training exercise for data extraction, where we achieved a high agreement of 94% between reviewers, we adjusted our approach for efficiency and feasibility. We decided to proceed with a second check for data extraction for the remaining studies due to this high level of agreement.

## Results

### Study selection

**Fig. 1:**
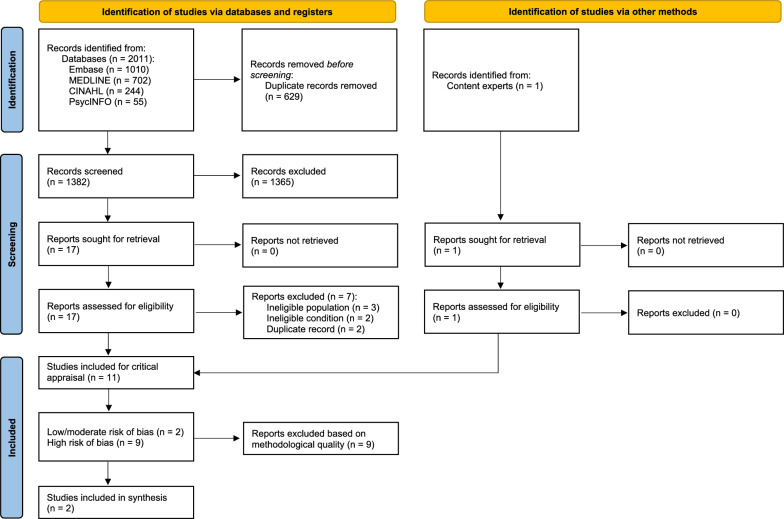
Preferred Reporting Items for Systematic reviews and Meta-Analyses flow diagram for study selection

Our search identified 2011 citations (Fig. 1) and one additional citation was identified through a content expert. We removed 629 duplicates and screened 1383 citations. Of those, 18 full-text articles were screened in phase two, and we identified 11 relevant studies [10–13, 16, 32–37]. The primary reasons for excluding seven articles were ineligible population (n = 3), ineligible condition (n = 2), and duplicate record (n = 2) (Fig. 1). The inter-rater agreement for Phase I screening was kappa = 0.71 (95% CI 0.52, 0.90) with a percent agreement of 99.4%. The inter-rater agreement for Phase II screening was kappa = 0.90 (95% CI 0.72, 1.0) and the percent agreement was 94.1%. Each of the 11 relevant studies reported prevalence estimates and two [35, 36] investigated factors associated with LBP. The inter-rater agreement for individual critical appraisal items was 82% (94/115 items across JBI Checklists), and 91% agreement on the overall methodological quality of studies.

## Risk of bias within studies

Of the 11 studies included, one had a low risk of bias [16], one had a moderate risk of bias [10], nine were rated as high risk of bias (see Table 1 and 2) [11–13, 32–37]. However, upon review of the draft manuscript, co-investigators disagreed with the final risk of bias assessment of one high risk of bias study. The disagreement focused on the participation rate of 11.8% and the likelihood that the study selection suffered from selection bias [11]. This will be further addressed in the following paragraphs.

**Table 1:** Risk of bias assessment for eligible prevalence studies using the JBI checklist for prevalence studies

Author, Year	1.1	1.2	1.3	1.4	1.5	1.6	1.7	1.8	1.9	Overall risk of bias
Bisiacchi (2006) [32]	UC	UC	UC	UC	UC	N	UC	UC	N	High
Campbell (2023) [16]	Y	Y	UC	Y	Y	Y	Y	Y	Y	Low
Hansen (2018) [10]	Y	Y	Y	Y	UC	UC	Y	UC	Y	Moderate
Holm (2006) [37]	Y	Y	Y	N	UC	UC	Y	UC	N	High
Howarth (2020) [11]	Y	Y	Y	Y	N	Y	Y	Y	N	High
Kizhakkeveettil (2014) [33]	Y	UC	UC	N	UC	UC	UC	UC	UC	High
Lamprecht (2019) [13]	Y	Y	UC	N	UC	N	Y	UC	Y	High
Macanuel (2005) [34]	Y	UC	UC	N	N	UC	UC	UC	Y	High
Mior (1987) [12]	Y	Y	Y	N	UC	UC	Y	UC	Y	High
Ndetan (2009) [35]	Y	UC	UC	N	Y	UC	Y	UC	Y	High
Ndetan (2009) [36]	Y	UC	UC	N	Y	UC	Y	UC	Y	High

Y, Yes; N, No; UC, Unclear; 1.1 Sample Frame: Was the sample frame appropriate to address the target population?; 1.2 Sample recruitment: Were study participants recruited in an appropriate way?; 1.3 Sample Size: Was the sample size adequate?; 1.4 Study Setting/Setting: Were the study subjects and setting described in detail?; 1.5 Coverage of sample: Was data analysis conducted with sufficient coverage of the identified sample?; 1.6 Identification of the condition: Were valid methods used for the identification of the condition?; 1.7 Condition measured: Was the condition measured in a standard, reliable way for all participants?; 1.8 Statistical Analysis: Was there appropriate statistical analysis?; 1.9 Response rate: Was the response rate adequate, and if not, was the low response rate managed appropriately?

**Table 2:** Risk of bias assessment for eligible cross-sectional analytic studies using the JBI checklist

Author, Year	1.1	1.2	1.3	1.4	1.5	1.6	1.7	1.8	Overall risk of bias
Ndetan (2009) [35]	Y	UC	UC	UC	UC	UC	UC	N	High
Ndetan (2009) [36]	Y	UC	UC	UC	UC	UC	UC	N	High

Y, Yes; N, No; UC, Unclear; 1.1 Inclusion criteria: Were the criteria for inclusion in the sample clearly defined?; 1.2 Subjects and setting: Were the study subjects and the setting described in detail?; 1.3 Measurement of exposure: Was the exposure measured in a valid and reliable way?; 1.4 Criteria for condition: Were objective, standard criteria used for measurement of the condition?; 1.5 Confounding factors: Were confounding factors identified?; 1.6 Strategies for Confounding: Were strategies to deal with confounding factors stated?; 1.7 Outcome measurement: Were the outcomes measured in a valid and reliable way?; 1.8 Statistical analysis: Was appropriate statistical analysis used?

The methodological weaknesses of the high risk of bias prevalence studies (Table 1) included failure to describe sampling methods [32–36], study subjects and setting [12, 13, 32–37] in detail, insufficient coverage of the sample [11–13, 32–37], unclear validity in the methods to identify low back pain (LBP) [13, 32–37], unclear reliability for the measurement of LBP [32–34], unclear statistical analysis [12, 13, 32–37], and inadequate response rate or failure to manage non-responder bias [11, 13, 32, 33, 37]. The methodological weaknesses of the high risk of bias studies with analytical components (Table 2) included failure to clearly describe the exposure measurement method, failure to identify important confounders and strategies to deal with confounding, unclear validity and reliability in the measurement of LBP, and inappropriate statistical analysis [35, 36].

The strengths of the study with low risk of bias included clear description of the sample frame, sampling methods, study subjects and setting, sufficient coverage of the sample, reliability and validity of the measurement of the condition, statistical analysis, and a 67% response rate with non-responder analysis [16]. Hansen et al. (2018) was rated as moderate risk of bias due to several factors: unclear definition of the outcome, lack of reporting of confidence estimates, and absence of a non-responder analysis, which puts it at risk for measurement bias and coverage bias. However, its strengths were that it conducted a national survey with a 65.2% response rate and provided clear descriptions of the sample frame, sample recruitment, study subjects, setting, and measurement of the condition. Therefore, our qualitative synthesis includes one low risk of bias study and one moderate risk of bias study. Authors of studies that included unclear ratings for specific items were contacted for clarification; however, only one author response was received from Howarth et al.

As per protocol, we did reach consensus on the overall risk of bias of the study by Howarth et al. (2020) [11]. Two reviewers independently appraised the study but disagreed on its overall risk of bias. A third reviewer appraised the paper, and after discussion, reviewers reached consensus rating the study as high risk of bias due to the low response rate (11.8%) and high probability of selection bias due to differences in the sample characteristics compared to those of the source population, particularly in age, years of practice, and location. Upon review of the draft manuscript by all authors, disagreements were raised on the risk of bias rating attributed to the study by Howarth et al. (2020). Despite thorough discussion, consensus was not reached. Therefore, we elected to conduct a post-hoc sensitivity analysis that includes the result reported in Howarth et al. (2020).

## Study characteristics

Both the low and moderate risk of bias studies were cross-sectional studies reporting on prevalence (see Table 3) [10, 16]. One study investigated chiropractors [10], and one studied chiropractic students [16].

**Table 3:** Descriptive cross-sectional studies of the prevalence of LBP in chiropractors and chiropractic students

Author(s), Year	Setting and target population	Sample size (No), response rate (%)	Age (years), mean (SD) or %	Sex (% female)	Years in practice, mean (SD) or %, or Year of Study, %	Low back pain definition	Prevalence (95% CI)
*Chiropractors*							
Hansen et al. (2018) [10]	Chiropractors practicing in primary care chiropractic clinics in Denmark and who were members of the Danish Chiropractors’ Association in August 2016	376, 65.2	43 (NR)	58.3	15 (NR)	Work-related acute physical injuries to the low back in the past year	1-year (acute): Women 3.4% (1.6, 6.8)^‡^; Men 0.7% (0.1, 3.7)^‡^
						Work-related overuse complaints in the low back in the past year	1-year (overuse): Women 35.6% (29.1, 42.0)^‡^; Men 22.4% (16.3, 29.6)^‡^
*Chiropractic Students*							
Campbell et al. (2023) [16]	Post-secondary students aged 18 years or older attending the Canadian Memorial Chiropractic College between October and November 2017, in Ontario, Canada	510, 67	24.6 (2.7)	60%	Year 1: 25.7%, Year 2: 26.9%, Year 3: 29.2%, Year 4: 18.2%	Any LBP in the past 7 days, defined as pain between the lower costal margins and the inferior gluteal folds (shaded body diagram)	1-week (any): Overall 69% (64.8, 73.0); Female 72.5% (67.1, 77.4); Male 64% (57.0, 70.6)
						LBP that is ≥ 3/10 on the 11-point NRS, defined as pain between the lower costal margins and the inferior gluteal folds	1-week (≥ 3/10): Overall 47.8% (43.4, 52.2); Female 51.6% (45.8, 57.3); Male 42.3% (35.4, 49.4)

NR, not reported; LBP, low back pain; CI, confidence interval; NRS, Numeric Rating Scale; ‡ Confidence interval for proportions calculated by investigators using the Wilson score method without continuity correction [31]

*Chiropractors.* The study of chiropractors was conducted in Denmark [10] and surveyed all chiropractors practicing in primary care chiropractic clinics and who were members of the Danish chiropractic association. A total of 376 chiropractors participated (response rate = 65.2%). The mean age of participants was 43 years and 58.3% were female. On average, participants had been in practice for 15 years. They evaluated the 12-month prevalence of work-related overuse injuries and acute complaints to the low back [10]. The authors did not define overuse or acute low back pain.

*Chiropractic students.* Campbell et al. (2023) surveyed a sample of 510 students enrolled (response rate = 67%) at the Canadian Memorial Chiropractic College (CMCC) in 2017. The mean age of the sample was 24.5 (SD 2.7) years and 60% were female. They evaluated the 7-day prevalence of LBP, where LBP was defined as the region between the lower costal margin and inferior gluteal fold [16].

## Findings from cross-sectional studies

*Chiropractors.* Among chiropractors practicing in primary care clinics in Denmark, the 12-month prevalence of work-related acute physical injuries to the low back was 3.4% (95% CI 1.6, 6.8) for women, and 0.7% (95% CI 0.1, 3.7) for men [10]. The 12-month prevalence of work-related overuse complaints in the low back was 35.6% (95% CI 29.1, 42.0) for women and 22.4% (95% CI 16.3, 29.6) for men.

*Chiropractic Students.* The 1-week prevalence of low back pain among CMCC chiropractic students was 69.0% (95% CI 64.8, 73.0) [16]. The 1-week prevalence varied by gender, where it was 72.5% (95% CI 67.1, 77.4) in females and 64% (95% CI 57.0, 70.6) in males. The 1-week prevalence of low back that is ≥ 3/10 on the 11-point Numeric Rating Scale (NRS) was 47.8% (95% CI 43.4, 52.2). In females, the 1-week prevalence for LBP that is ≥ 3/10 on the 11-point NRS was 51.6% (95% CI 45.8, 57.3), and in males it was 42.3% (95% CI 35.4, 49.4).

## Post-hoc sensitivity analysis

*Study Characteristics.* The source population for the cross-sectional study by Howarth et al. included all members of the Ontario Chiropractic Association (see Table 4) [11]. The age of chiropractors ranged from 25 to 70 + years, and 39.3% were females. The mean patient contact hours per week was 28 hours. The study investigated work-related lower back pain in the previous year. Overall, 432 participated for a participation rate of 11.8%.

**Table 4:** Post-hoc sensitivity analysis

Author(s), Year	Setting and target population	Sample size (No), response rate (%)	Age (years), mean (SD) or %	Sex (% female)	Years inpractice, mean (SD) or %, or Year of study, %	Low back pain definition	Prevalence (95% CI)
*Chiropractors*							
Howarth et al. (2020) [11]	Chiropractors who were members of the Ontario Chiropractic Association between January and March 2019, in Ontario, Canada	432, 11.8%	25–29: 10%, 30–34: 13.2%, 35–39: 13.4%, 40–44: 13.7%, 45–49: 18.1%, 50–54: 9.0%, 55–59: 6.9%, 60–64: 7.9%, 65–69: 6.3%, 70 + : 1.2%	39.3%	0–1: 5.3%, 2–5: 11.8%, 6–10: 14.6%, 11–20: 30.6%, 21–30: 19.2%, 31–40: 14.6%, 40 + : 3.2%	Work-related musculoskeletal trouble in lower back in the past year, measured using the Standardized Nordic Musculoskeletal Questionnaire (shaded body diagram)	1-year: 38.3% (36.1, 40.6)

SD, standard deviation; CI, confidence interval

*Findings*. The 12-month prevalence of work-related musculoskeletal trouble in the lower back was 38.3% (95% CI 36.1, 40.6) among chiropractors in Ontario, Canada [11].

The sensitivity analysis reveals the burden of LBP among chiropractors in Canada, demonstrating similarities to the 12-month prevalence of work-related overuse injuries in Danish chiropractic women, and slightly higher than that among men. The sensitivity analysis does not significantly change the systematic review’s overall findings.

## Discussion

### Summary of evidence

Our systematic review aimed to synthesize the evidence on the epidemiology of LBP in chiropractors and chiropractic students. The evidence from one moderate risk of bias prevalence study suggests that chiropractors primarily report work-related overuse injuries to the low back (35.6%, 95% CI 29.1, 42.0 in women; 22.4%, 95% CI 16.3, 29.6 in men), and less commonly report work-related acute injuries (3.4%, 95% CI 1.6, 6.8 in women; 0.7%, 95% CI 0.1, 3.7 in men) [10]. Compared to previous literature, this was lower than the overall prevalence of LBP reported in physiotherapists globally (40.1%, [95% CI: 32.2, 48.0], unclear period) [9], and lower than the 12-month prevalence of work-related LBP in physiotherapists in France (41.7% among women and 37.4% among men) [38]. However, due to unclear case definitions, direct comparisons are challenging [10]. Moreover, one low risk of bias study found a high one-week prevalence of LBP among chiropractic students (69.0%, [95% CI 64.8, 73.0]) [16]. When compared to other health professions, this prevalence was higher than the one-week prevalence found in nursing (28.0%, [95% CI 24.0, 32.0]) and medical students (34.0%, [95% CI 7.0, 62.0]) students [21], as well as physiotherapy students in Australia (27.6%, [95% CI 21.9, 33.2]) [17]. Both studies included in our review reported that LBP was more prevalent among females [10, 16].

To our knowledge, this is the first systematic review investigating the epidemiology of LBP among chiropractors and chiropractic students. We did not find any high-quality studies investigating factors associated with LBP, the incidence of LBP, or the risk factors of LBP in chiropractors or chiropractic students. Our review highlights the importance of addressing LBP while training to become a chiropractor, as well as throughout one’s career. Our findings also align with the 2019 Global Burden of Disease Study, which showed a higher prevalence of LBP among females [39]. This underscores the importance of investigating the factors associated with LBP in both males and females to better understand the differences in etiology related to sex.

## Strengths and limitations

Our review has several strengths. Our protocol was described a priori and registered on Open Science Framework. Our review used the reporting guidelines from PRISMA and the SWiM checklist for reporting narrative syntheses, and we used JBI critical appraisal tools to assess the quality of the evidence [20, 27–29]. Furthermore, we did not have restrictions on language in our search to make sure we retrieved all possibly relevant studies. However, we did not identify any possibly relevant articles in a non-English language. Finally, we only included studies that were low to moderate risk of bias in our synthesis, which improves the certainty of our findings.

Our review has limitations. First, we did not search for grey literature in our search strategy, which could increase the risk of publication bias. Second, the critical appraisal varied among the reviewers for one study, highlighting the subjectivity and challenges in risk of bias assessments. However, we followed a rigorous consensus process to minimize this problem and decided to conduct a post-hoc sensitivity analysis after thorough discussion. Third, we did not assess the certainty of the evidence using the GRADE approach, as factors such as inconsistency and indirectness are less relevant to our review. Since we did not conduct a meta-analysis and are focusing on direct evidence related to predefined questions, we opted not to apply the GRADE framework. However, we do address three GRADE domains throughout our review: 1) risk of bias, 2) precision, and 3) publication bias.

## Implications and future research

We found evidence that LBP is prevalent among chiropractors and chiropractic students. This finding suggests that future research is needed to enhance our understanding of the etiology of LBP in these populations. Studies should investigate associated factors to gain a deeper insight of the etiology to LBP in this population. Furthermore, we were unable to address our second objective regarding the incidence and risk factors of LBP due to the absence of relevant published longitudinal cohort studies. This highlights the need for more high-quality cohort studies on LBP in chiropractors and chiropractic students.

Nine studies were excluded from our synthesis due to high risk of bias which highlights the need to improve the methodology of epidemiological research in this field. Researchers should consider providing more detail when reporting methodological components of their studies. This includes a clear description of the study sample, such as demographics, comorbidities, and other potential influential factors. Additionally, researchers should describe the setting in detail, including contextual factors, so researchers can determine if it is comparable to the population of interest. Recruitment methods need thorough description, as this can impact the representativeness of the sample. Furthermore, researchers should clearly define their outcomes, as unclear definitions can lead to measurement bias. They should use valid and reliable instruments to measure their outcomes and covariates, and, when possible, provide the survey instrument used in the study. Finally, evaluating non-responder analyses is essential to understand any potential influence of selection bias. Investigators should be encouraged to use a reporting guideline such as Strengthening the Reporting of Observation studies in Epidemiology (STROBE) [40].

## Conclusion

Our systematic review included two prevalence studies of low to moderate risk of bias investigating the prevalence of LBP among chiropractors and chiropractic students. We found evidence that chiropractors and chiropractic students commonly report LBP. Our review highlights the need for future high-quality studies in this population. Future research should investigate the factors or risk factors associated with LBP to better understand the etiology in this population.

## Supplementary Information

Additional file 1.

Additional file 2.

## Data Availability

Not applicable.

## List of Abbreviations

CI: Confidence interval
CMCC: Canadian Memorial Chiropractic College
JBI: Joanna Briggs Institute
LBP: Low back pain
NRS: Numeric rating scale
PRESS: Peer review of electronic search strategies
PRISMA: Preferred reporting items for systematic reviews and meta-analyses
SD: Standard deviation
STROBE: Strengthening the reporting of observational studies in epidemiology
SWiM: Synthesis without meta-analysis
