# Supplementary material for: The epidemiology of low back pain in chiropractors and chiropractic students: a systematic review of the literature
Source: Chiropr Man Therap. 2024 Nov 26;32:36. doi: 10.1186/s12998-024-00559-8 (PMC11590469; doi:10.1186/s12998-024-00559-8)
Supplement: Supplementary file 2 — Additional file 2. [file 12998_2024_559_MOESM2_ESM.pdf]

Additional File 2 – Search Strategy

1 – Search Strategy

Database & Platform: Ovid MEDLINE

Investigator: Dr Lauren Ead

Librarian: Kent Murnaghan

Years of search: Inception - current **Languages**: No limit

Date search run: MAY 1 2023 **Number of records retrieved: 704**

Search Strategy:

1. Low Back Pain/
2. exp Back Pain/
3. exp Back Injuries/
4. Intervertebral Disc Degeneration/
5. Intervertebral Disc Displacement/
6. Osteoarthritis, Spine/
7. Piriformis Muscle Syndrome/
8. Polyradiculopathy/
9. Sciatica/
10. Spinal Diseases/
11. Spinal Stenosis/
12. Spinal Injuries/
13. Spondylolysis/
14. Osteoarthritis/
15. Synovial Cyst/
16. Spinal Curvatures/
17. Lumbar Vertebrae/ in
18. Lumbosacral region/ in
19. exp Back Muscles/in
20. Coccyx/in
21. Zygaphophyseal Joint/in
22. Intervertebral Disc/in
23. Lumbosaccral Plexus/in
24. Sacroiliac Joint/in
25. (low* adj2 (back adj2 (pain* or facet or (nerve adj2 root*) or osteoarth* or radicul* or stenos* or spondylo* or injur* or discomfort* or dysfunction* or sore* or herniat* or trauma* or sprain* or strain* or ach*))).mp.
26. (low* adj2 (trunk* adj2 (pain* or facet or (nerve adj2 root*) or osteoarth* or radicul* or stenos* or spondylo* or injur* or discomfort* or dysfunction* or sore* or herniat* or trauma* or sprain* or strain* or ach*))).mp.
27. (low* adj2 (back-pain* or back-injur* or trunk-pain*)).mp.

28. (lumbar* adj3 (pain* or facet or (nerve adj2 root*) or osteoarth* or radicul* or stenos* or spondylo* or zygapophys* or injur* or discomfort or dysfunction* or sore* or herniat* or trauma* or sprain* or strain* or ach*)).mp.
29. lumbo* adj3 (pain* or facet or (nerve adj2 root*) or osteoarth* or radicul* or stenos* or spondylo* or zygapophys* or injur* or discomfort or dysfunction* or sore* or herniat* or trauma* or sprain* or astrain* or ach*).mp.
30. (lumbar* adj3 (disc* adj3 (extru* or degenerat* or displac* or herniat* or prolaps* or sequestered* or slipped* or protru* or avuls*))).mp.
31. (lumbar* adj3 (disk* adj3 (extru* or degenerat* or displac* or herniat* or prolaps* or sequestered* or slipped* or protru* or avuls*))).mp.
32. (back adj2 (pain* or facet or (nerve adj2 root*) or osteoarth* or radicul* or stenos* or spondylo* or injur* or discomfort* or dysfunction* or sore* or herniat* or trauma* or sprain* or strain* or ach*))).mp.
33. back-pain*.mp.
34. (backach* or back-ach*).mp.
35. (back adj2 muscle*) adj2 pain*.mp.
36. (intervertebral* adj3 (disc* adj3 (extru* or degenerat* or displac* or herniat* or prolaps* or sequestered or slipped or protru* or avuls*))) .mp.
37. (intervertebral* adj3 (disk* adj3 (extru* or degenerat* or displac* or herniat* or prolaps* or sequestered or slipped or protru* or avuls*))) .mp.
38. (vertebral* adj3 (disc* adj3 (extru* or degenerat* or displac* or herniat* or prolaps* or sequestered or slipped or protru* or avuls*))) .mp.
39. (vertebral* adj3 (disk* adj3 (extru* or degenerat* or displac* or herniat* or prolaps* or sequestered or slipped or protru* or avuls*))) .mp.
40. coccydyn*.mp.
41. (coccyx* adj2 (pain* or facet or (nerve adj2 root*) or osteoarth* or radicul* or stenos* or spondylo* or zygapophys* or injur* or discomfort* or dysfunction* or sore* or herniat* or sprain* or strain* or trauma*)).mp.
42. (coccygodyn* or coccalg*).mp.
43. coccygalg*.mp.
44. coccygeal* adj2 (pain* or facet or (nerve adj2 root*) or osteoarth* or radicul* or stenos* or spondylo* or zygapophys* or injur* or discomfort* or dysfunction* or sore* or herniat* or sprain* or strain* or trauma*).mp.
45. dorsalg*.mp.
46. lumbago*.mp.
47. lumboischialg*.mp.
48. piriformis* adj2 syndrome*.mp.
49. sacral* adj3 (pain* or facet or nerve adj2 root* or osteoarth* or radicul* or stenos* or spondylo* or zygapophys* or injur* or discomfort or dysfunction* or sore* or herniat* or sprain* or strain* or trauma* or ach*).mp.
50. sacro* adj3 (pain* or facet or nerve adj2 root* or osteoarth* or radicul* or stenos* or spondylo* or zygapophys* or injur* or discomfort or dysfunction* or sore* or herniat* or sprain* or strain* or trauma* or ach*).mp.
51. sacrum* adj2 (pain* or facet or (nerve adj2 root*) or osteoarth* or radicul* or stenos* or spondylo* or zygapophys* or injur* or discomfort or dysfunction* or sore* or herniat* or sprain* or strain* or trauma* or ach*).mp.

52. SI adj2 (joint* adj2 (pain* or facet or (nerve adj2 root*) or osteoarth* or radicul* or stenos* or spondylo* or zygapophys* or injur* or discomfort or dysfunction* or sore* or herniat* or sprain* or strain* or ach*)).mp.
53. sciatic*.mp.
54. stenos* adj2 (spine* or spinal* or vertebral*).mp.
55. (spine or spinal) adj2 osteoarthr*.mp.
56. spine* adj3 (condition* or diseas* or disab* or disorder* or degen* or pain* or stenos*).mp.
57. spinal* adj3 (condition* or diseas* or disab* or disorder* or degen* or pain* or stenos*).mp.
58. spondylosis*.mp.
59. tailbone* adj3 (pain* or facet or (nerve adj2 root*) or osteoarth* or radicul* or stenos* or spondylo* or injur* or discomfort* or dysfunction* or sore* or herniat* or sprain* or strain* or ach*)).mp.
60. vertebr* adj3 (pain* or facet or (nerve adj2 root*) or osteoarth* or radicul* or stenos* or spondylo* or injur* or discomfort* or dysfunction* or sore* or herniat* or sprain* or strain* or ach*)).mp
61. (poly-radicul* or polyradicul*).mp.
62. neuropath* adj2 (lumbar* or lumbo* or sacral* or sacro* or (low* adj2 back) or low-back* or lower-back* or spine* or spinal* or L1 or L2 or L3 or L4 or L5).mp.
63. radiculopath* adj3 (lumbar* or lumbo* or sacral* or sacro* or (low* adj2 back) or low-back* or lower-back* or spine* or spinal* or L1 or L2 or L3 or L4 or L5).mp.
64. radiating* adj3 (lumbar* or lumbo* or sacral* or sacro* or (low* adj2 back) or low-back* or lower-back* or spine* or spinal* or L1 or L2 or L3 or L4 or L5).mp.
65. radicular* adj3 (lumbar* or lumbo* or sacral* or sacro* or (low* adj2 back) or low-back* or lower-back* or spine* or spinal* or L1 or L2 or L3 or L4 or L5).mp.
66. (lumborum* or longissimus* or (erector* adj2 spin*)).mp.
67. synovial* adj2 cyst*.mp.
68. thoracolumbar* adj2 (pain* or facet* or nerve* adj2 root* or osteoarthr* or radicul* or stenos* or spondylo* or injur* or trauma* or discomfort* or dysfunction* or sore* or herniat* or sprain* or strain* or trauma* or ach*).mp.
69. thoraco-lumbar* adj2 (pain* or facet* or nerve* adj2 root* or osteoarthr* or radicul* or stenos* or spondylo* or zygapohys* or injur* or trauma* or discomfort* or dysfunction* or sore* or herniat* or sprain* or strain* or trauma* or ach*).mp.
70. curvatur* adj2 (spine* or spinal*).mp.
71. (pathol* adj2 (lumbar* or (low* adj2 back) or low-back* or (lower* adj2 back) or lower-back* or thoracolumbar* or thoraco-lumbar* or intervertebral* or lumbosacral* or lumbo-sacral* or sacral* or sacro-iliac* or sacroiliac*)).mp.

72. Occupational Injuries/
73. Wounds and Injuries/
74. Occupational Diseases/
75. Musculoskeletal System/
76. Musculoskeletal Diseases/
77. Neuromuscular Diseases/

78. (disease* or injur* or disorder* or complaint*) adj2 (work* or occupation* or musculoskeletal* or neuromuscul* or overuse*).mp.
79. repetitive* adj2 (sprain* or strain*).mp.
80. OR /1-79 [* Low Back Pain]**

81. Students/
82. (student* or trainee* or intern or interns or residen*) .mp.
83. Chiropractic/
84. chiropr*.mp.
85. chiropractor*.mp.
86. (doctor* or physician* or practitioner* or clinician* or practice* or provider*).mp.
87. (doctor* or physician* or practitioner* or clinician* or practice* or provider*) adj2 chiropr*).mp.
88. or/81-82
89. or/83-84
90. 83 AND 86
91. 88 AND 89
92. OR/ 85 or 87 or 90 or 91 [* Chiropractors/ Chiropractic Students]**

93. Incidence/
94. Prevalence/
95. incidence*.mp.
96. prevalen*.mp.
97. occurrence* .mp.
98. rate*.mp.
99. proportion*.mp.
100. first onset.mp.
101. first episode.mp.
102. OR /93-101 [* Incidence/ Prevalence]**

103. Risk Factors/
104. Sociodemographic Factors/
105. Social Environment/
106. Mental Health/
107. Stress, Psychological/
108. exp Comorbidity/
109. exp Chronic Disease/
110. exp Heart Diseases/
111. exp Stroke/
112. exp Neoplasms /
113. exp Diabetes Mellitus/
114. Workplace/psychology
115. exp Exercise/
116. Sedentary Behavior/
117. Substance-Related Disorders/

118. Sleep Quality/
119. Occupational Exposure/
120. Posture/
121. ((risk* adj2 factor*) or (associated* adj2 factor*)).mp.
122. (comorbid* OR (chronic* adj2 disease*) OR (heart* adj2 disease*) or stroke* or cancer* or tumour* or tumor* or neoplas* or metast* or malignan* OR diabet*).mp.
123. health* adj2 correlat*.mp.
124. ((sociodemographic* adj2 factor*) or (socio-demographic* adj2 factor*) or sex or gender* or income or (employment* adj2 status*) or (social* adj2 environment*)) .mp.
125. ((psychological* adj2 distress*) or (mental* adj2 distress*) or stress* or anxiety* or anxious* or depress* or (mental* adj2 health*) or psychosocial* or psycho-social*).mp.
126. ((physical* adj2 activit*) or (resistance* adj2 training*) or (resistance* adj2 activit*) or (aerobic* adj2 training*) or (aerobic* adj2 activit*) or (endurance* adj2 training*) or (endurance* adj2 activit*) or (strength* adj2 training*) or (strength* adj2 activit*) or (activit* adj2 level*) or exercis*).mp.
127. ((sedentary* adj2 behavior*) or (sedentary* adj2 behaviour*) or (physical* adj2 inactivit*)).mp.
128. ((substance-related* adj2 disorder*) or (drug* adj2 addict*) or (alcohol* adj2 abus*) or (alcohol* adj2 addict*) or (drug* adj2 abus*) or (drug* adj2 depend*) or substance-use* or (substance* adj2 use*)).mp.
129. ((sleep* adj2 qualit*) or (sleep* adj2 hygien*)).mp.
130. ((occupational* adj2 exposur*) or (physical* adj2 exposur*) or postur* or (perform* adj3 (manip* or adjust*)) or position*) .mp.

131. OR /103-130 [**Risk Factors]

132. Survey and Questionnaires/
133. Cross-Sectional Studies/
134. exp Cohort Studies/
135. Epidemiologic Studies/
136. (survey* or questionnair* or cross-section* or (cross* adj1 section*) or cohort* or (observational* adj2 (study or studies or analy*)) or follow-up* or prospective* or retrospectiv* or longitudinal*) .mp.
137. observational study.pt.

138. **OR /132-137 [** Study Design]**

139. 80 and 92 and (102 or 131 or 138)
[LBP and Chiropractors/Chiro Students and (Prevalence OR Risk Factors OR Study Design)]

Database & Platform: CINAHL

Investigator: Dr Lauren Ead

Librarian: Kent Murnaghan

Years of search: Inception - current **Languages**: No limit

Date search run: MAY 1 2023 **Number of records retrieved:** **244**

Search Strategy:

1. MH Low Back Pain
2. MH Back Pain+
3. MH Back Injuries
4. MH Intervertebral Disc Displacement
5. MH Intervertebral Disc/IN
6. MH Lumbar Vertebrae/IN
7. MH Lumbosacral Plexus/IN
8. MH Coccyx/IN
9. MH Osteoarthritis, Spine
10. MH Osteoarthritis
11. MH Piriformis Muscles/IN
12. MH Polyradiculopathy/
13. MH Sacroiliac Joint/IN
14. MH Sciatica
15. MH Spinal Curvatures
16. MH Spinal Diseases
17. MH Spinal Injuries
18. MH Spinal Stenosis
19. MH Spondylolysis+
20. MH Spondylosis
21. MH Synovial Cyst
22. MH Zygaphophyseal Joint/IN
23. TI ((low* n2 (back n2 pain*)) or (low-back* n2 pain*) or (lower-back* n2 pain*) or (low* n2 back-pain*)) or AB ((low* n2 (back n2 pain*)) or (low-back* n2 pain*) or (lower-back* n2 pain*) or (low* n2 back-pain*))
24. TI ((low* n2 (back n2 injur*)) or (low-back* n2 injur*) or (lower-back* n2 injur*) or (low* n2 back-injur*)) or AB ((low* n2 (back n2 injur*)) or (low-back* n2 injur*) or (lower-back* n2 injur*) or (low* n2 back-injur*))
25. TI ((low* n2 (back n2 trauma*)) or (low-back n2 trauma*) or (lower-back* n2 trauma*) or (low* n2 back-trauma*)) or AB ((low* n2 (back n2 trauma*)) or (low-back n2 trauma*) or (lower-back* n2 trauma*) or (low* n2 back-trauma*))
26. TI ((low* n2 (trunk n2 pain*)) or (lower-trunk* n2 pain*) or (low* n2 trunk-pain*)) or AB ((low* n2 (trunk n2 pain*)) or (lower-trunk* n2 pain*) or (low* n2 trunk-pain*))
27. TI lumbar* n3 (disc* n3 (extru* or degenerat* or displac* or herniat* or prolaps* or sequestered or slipped or protru* or avuls*)) or AB lumbar* n3 (disc* n3 (extru* or degenerat* or displac* or herniat* or prolaps* or sequestered or slipped or protru* or avuls*))
28. TI lumbar* n3 (disk* n3 (extru* or degenerat* or displac* or herniat* or prolaps* or sequestered or slipped or protru* or avuls*)) or AB lumbar* n3 (disk* n3 (extru* or degenerat* or displac* or herniat* or prolaps* or sequestered or slipped or protru* or avuls*))
29. TI lumbar* n3 (pain* or facet* or (nerve n2 root*) or osteoarth* or radicul* or stenos* or spondylo* or zygapophys* or injur* or discomfort* or dysfunction* or sore* or herniat*)

or AB lumbar* n3 (pain* or facet* or (nerve n2 root*) or osteoarth* or radicul* or stenos* or spondylo* or zygapophys* or injur* or discomfort* or dysfunction* or sore* or herniat*)

30. TI lumbo* n3 (pain* or facet* or (nerve n2 root*) or osteoarth* or radicul* or stenos* or spondylo* or zygapophys* or injur* or discomfort* or dysfunction* or sore* or herniat*) or AB lumbo* n3 (pain* or facet* or (nerve n2 root*) or osteoarth* or radicul* or stenos* or spondylo* or zygapophys* or injur* or discomfort* or dysfunction* or sore* or herniat*)
31. TI back n3 (ach* or injur* or pain* or sprain* or strain* or disorder*) or AB back n3 (ach* or injur* or pain* or sprain* or strain* or disorder
32. TI backach* or AB backach*
33. TI back-pain* or AB back-pain*
34. TI intervertebral* n3 (disc* n3 (extru* or degenerat* or displac* or herniat* or prolaps* or sequestered or slipped or protru* or avuls*)) or AB intervertebral* n3 (disc* n3 (extru* or degenerat* or displac* or herniat* or prolaps* or sequestered or slipped or protru* or avuls*))
35. TI intervertebral* n3 (disk* n3 (extru* or degenerat* or displac* or herniat* or prolaps* or sequestered or slipped or protru* or avuls*)) or AB intervertebral* n3 (disk* n3 (extru* or degenerat* or displac* or herniat* or prolaps* or sequestered or slipped or protru* or avuls*))
36. TI coccy* n2 (ach* or injur* or pain* or sprain* or strain*) or AB coccy* n2 (ach* or injur* or pain* or sprain* or strain*)
37. TI (coccygodyn* or coccalg* or coccygalg*) or AB (coccygodyn* or coccalg* or coccygalg*)
38. TI dorsalg* or AB dorsalg*
39. TI lumbago* or AB lumbago*
40. TI lumboischialg* or AB lumboischialg*
41. TI (piriformis* n2 syndrome*) or AB (piriformis* n2 syndrome*)
42. TI sacral* n3 (pain* or facet* or (nerve n2 root*) or osteoarth* or radicul* or stenos* or spondylo* or zygapophys* or injur* or discomfort* or dysfunction* or sore* or herniat*) or AB sacral* n3 (pain* or facet* or (nerve n2 root*) or osteoarth* or radicul* or stenos* or spondylo* or zygapophys* or injur* or discomfort* or dysfunction* or sore* or herniat*)
43. TI sacro* n3 (pain* or facet* or (nerve n2 root*) or osteoarth* or radicul* or stenos* or spondylo* or zygapophys* or injur* or discomfort* or dysfunction* or sore* or herniat*) or AB sacro* n3 (pain* or facet* or (nerve n2 root*) or osteoarth* or radicul* or stenos* or spondylo* or zygapophys* or injur* or discomfort* or dysfunction* or sore* or herniat*)
44. TI "si" n2 (joint* n3 (pain* or facet* or (nerve n2 root*) or osteoarth* or radicul* or stenos* or spondylo* or zygapophys* or injur* or discomfort* or dysfunction* or sore* or herniat*)) or AB "si" n2 (joint* n3 (pain* or facet* or (nerve n2 root*) or osteoarth* or radicul* or stenos* or spondylo* or zygapophys* or injur* or discomfort* or dysfunction* or sore* or herniat*))
45. TI sacrococcy* n2 (ach* or injur* or pain* or sprain* or strain*) or AB sacrococcy* n2 (ach* or injur* or pain* or sprain* or strain*)

46. TI sacrum* n2 (ach* or injur* or pain* or sprain* or strain*) or AB sacrum* n2 (ach* or injur* or pain* or sprain* or strain*)
47. TI sciatic* or sciatic*
48. TI stenos* n2 (spine* or spinal* or vertebral*) or AB stenos* n2 (spine* or spinal* or vertebral*)
49. TI (spine* or spinal*) n2 osteoarthr* or AB (spine* or spinal*) n2 osteoarthr*
50. TI spine* n3 (condition* or diseas* or disabilit* or disorder* or degenerat* or pain* or stenos*) or AB spine* n3 (condition* or diseas* or disabilit* or disorder* or degenerat* or pain* or stenos*)
51. TI spinal* n3 (condition* or diseas* or disabilit* or disorder* or degenerat* or pain* or stenos*) or AB spinal* n3 (condition* or diseas* or disabilit* or disorder* or degenerat* or pain* or stenos*)
52. TI spondylo* or AB spondylo*
53. TI tailbone* n3 (ach* or injur* or pain* or sprain* or strain*) or AB tailbone* n3 (ach* or injur* or pain* or sprain* or strain*)
54. TI vertebr* n3 (ach* or injur* or pain* or sprain* or strain*) or AB vertebr* n3 (ach* or injur* or pain* or sprain* or strain*)
55. TI (poly-radicul* or polyradicul*) or AB (poly-radicul* or polyradicul*)
56. TI neuropath* n2 (lumbar* or lumbo* or sacral* or sacro* or (low* n2 back) or low-back* or lower-back* or spine* or spinal* or L1 or L2 or L3 or L4 or L5) or AB neuropath* n2 (lumbar* or lumbo* or sacral* or sacro* or (low* n2 back) or low-back* or lower-back* or spine* or spinal* or L1 or L2 or L3 or L4 or L5)
57. TI radiculopath* n3 (lumbar* or lumbo* or sacral* or sacro* or (low* n2 back) or low-back* or lower-back* or spine* or spinal* or L1 or L2 or L3 or L4 or L5) or AB radiculopath* n3 (lumbar* or lumbo* or sacral* or sacro* or (low* n2 back) or low-back* or lower-back* or spine* or spinal* or L1 or L2 or L3 or L4 or L5)
58. TI radiating* n3 (lumbar* or lumbo* or sacral* or sacro* or (low* n2 back) or low-back* or lower-back* or spine* or spinal* or L1 or L2 or L3 or L4 or L5) or AB radiating* n3 (lumbar* or lumbo* or sacral* or sacro* or (low* n2 back) or low-back* or lower-back* or spine* or spinal* or L1 or L2 or L3 or L4 or L5)
59. TI radicular* n3 (lumbar* or lumbo* or sacral* or sacro* or (low* n2 back) or low-back* or lower-back* or spine* or spinal* or L1 or L2 or L3 or L4 or L5) or AB radicular* n3 (lumbar* or lumbo* or sacral* or sacro* or (low* n2 back) or low-back* or lower-back* or spine* or spinal* or L1 or L2 or L3 or L4 or L5)
60. TI lumborum* n3 (ach* or injur* or pain* or sprain* or strain*) or AB lumborum* n3 (ach* or injur* or pain* or sprain* or strain*)
61. TI longissimus* n3 (ach* or injur* or pain* or sprain* or strain*) or AB longissimus* n3 (ach* or injur* or pain* or sprain* or strain*)
62. TI (erector n2 spin*) n3 (ach* or injur* or pain* or sprain* or strain*) or AB erector n2 spin*) n3 (ach* or injur* or pain* or sprain* or strain*)
63. TI synovial* n2 cyst* or AB synovial* n2 cyst*
64. TI thoracolumbar* n3 (pain* or facet* or (nerve* n2 root*) or osteoarthr* or radicul* or stenos* or spondylo* or zygapohys* or injur* or trauma* or discomfort* or dysfunction* or sore* or herniat*) or AB thoracolumbar* n3 (pain* or facet* or (nerve* n2 root*) or osteoarthr* or radicul* or stenos* or spondylo* or zygapohys* or injur* or trauma* or discomfort* or dysfunction* or sore* or herniat*)

65. TI thoraco-lumbar* n3 (pain* or facet* or (nerve* n2 root*) or osteoarthr* or radicul* or stenos* or spondylo* or zygapohys* or injur* or trauma* or discomfort* or dysfunction* or sore* or herniat*) or AB thoraco-lumbar* n3 (pain* or facet* or (nerve* n2 root*) or osteoarthr* or radicul* or stenos* or spondylo* or zygapohys* or injur* or trauma* or discomfort* or dysfunction* or sore* or herniat*)
66. TI curvatur* n2 (spine* or spinal*) or AB curvatur* n2 (spine* or spinal*)
67. TI (pathol* n2 (lumbar* or (low* n2 back) or low-back* or (lower* n2 back) or lower-back* or thoracolumbar* or thoraco-lumbar* or intervertebral* or lumbosacral* or lumbo-sacral* or sacral* or sacro-iliac* or sacroiliac*)) or AB (pathol* n2 (lumbar* or (low* n2 back) or low-back* or (lower* n2 back) or lower-back* or thoracolumbar* or thoraco-lumbar* or intervertebral* or lumbosacral* or lumbo-sacral* or sacral* or sacro-iliac* or sacroiliac*))

68. MH Occupational-Related injuries
69. MH Wounds and Injuries
70. MH Occupational diseases
71. MH Musculoskeletal system
72. MH Musculoskeletal diseases
73. "MH Neuromuscular diseases
74. TI disease* n2 (work* or occupation* or musculoskeletal* or neuromuscul* or overuse*) or AB disease* n2 (work* or occupation* or musculoskeletal* or neuromuscul* or overuse*)
75. TI injur* n2 (work* or occupation* or musculoskeletal* or neuromuscul* or overuse*) or AB injur* n2 (work* or occupation* or musculoskeletal* or neuromuscul* or overuse*) or TI repetitive* n2 (sprain* or strain*) or AB repetitive* n2 (sprain* or strain*)
76. TI disorder* n2 (work* or occupation* or musculoskeletal* or neuromuscul* or overuse*) or AB disorder* n2 (work* or occupation* or musculoskeletal* or neuromuscul* or overuse*)
77. TI complaint* n2 (work* or occupation* or musculoskeletal* or neuromuscul* or overuse*) or AB complaint* n2 (work* or occupation* or musculoskeletal* or neuromuscul* or overuse*)
78. **or/1-77 [** Low Back Pain]**

79. MH Students
80. (TI student OR AB student) OR (TI trainee* OR AB trainee*) or (TI intern or interns) or AB (intern or interns) or (TI resident* or AB resident*)
81. MH Chiropractic"
82. (TI chiropr* OR AB chiropr*)
83. MH Students, Chiropractic
84. MH Chiropractors
85. TI (doctor* or physician* or practitioner* or clinician* or practice* or provider*) or AB (doctor* or physician* or practitioner* or clinician* or practice* or provider*)

86. TI ((doctor* or physician* or practitioner* or clinician* or practice* or provider*) n2 chiropr*) or AB ((doctor* or physician* or practitioner* or clinician* or practice* or provider*) n2 chiropr*)
87. OR/ 79-80
88. OR/ 81-82
89. 87 AND 88
90. 81 AND 85
91. 83 or or 84 or 86 or 89 or 90 [** Chiropractors / Chiropractic Students]

92. MH Incidence
93. MH Prevalence
94. (TI incidence* OR AB incidence*)
95. (TI prevalen* OR AB prevalen*)
96. (TI "person-time rate*" OR AB "person-time rate*")
97. (TI occurrence* OR AB occurrence*)
98. (TI rate* OR AB rate*)
99. (TI proportion* OR AB proportion*)
100. (TI "first onset" OR AB "first onset")
101. (TI "first episode" OR AB "first episode")
102. OR/ 92-101 [** Incidence]

103. MH Risk Factors
104. MH Sociodemographic Factors
105. MH social Environment
106. MH Mental Health
107. MH Stress, Psychological
108. MH Comorbidity
109. MH Chronic Disease +
110. MH Heart Diseases +
111. MH Stroke +
112. MH Neoplasms +
113. MH Diabetes Mellitus +
114. MH Workplace
115. MH Occupational Exposure
116. MH Exercise
117. MH Lifestyle, Sedentary
118. MH Substance Use Disorders
119. MH Sleep Quality
120. MH Posture
121. TI (risk* or associated*) n2 factor* or AB (risk* or associated*) n2 factor*
122. TI health* n2 correlat* or AB health* n2 correlat*
123. TI (sociodemographic* or sex* or income* or (employment* n2 status*) or (social* n2 environment*)) or AB (sociodemographic* or sex* or income* or (employment* n2 status*) or (social* n2 environment*))

124. TI (mental* n2 health*) or (psycholog* n2 distress*) or depression* or anxiety* or stress* or (mental* n2 distress*) or psychosocial*) or AB (mental* n2 health*) or (psycholog* n2 distress*) or depression* or anxiety* or stress* or (mental* n2 distress*) or psychosocial*)
125. TI (exercis* or (physical* n2 activit*) or (resistance* n2 activit*) or (aerobic* n2 train*) or (aerobic* n2 activit*) or (endurance* n2 train*) or (endurance* n2 activit*) or (strength* n2 train*) or (strength* n2 activit*) or (activit* n2 level*)) or AB (exercis* or (physical* n2 activit*) or (resistance* n2 activit*) or (aerobic* n2 train*) or (aerobic* n2 activit*) or (endurance* n2 train*) or (endurance* n2 activit*) or (strength* n2 train*) or (strength* n2 activit*) or (activit* n2 level*))
126. TI ((sedentary* n2 behav*) or (physical* n2 inactivit*)) or AB ((sedentary* n2 behav*) or (physical* n2 inactivit*))
127. TI ((substance* n2 disorder*) or (drug* n2 addict*) or (abus* n2 alcohol*) or (addict* n2 alcohol*) or (abus* n2 drug*) or (drug* n2 depend*) or (substance* n2 abus*) or (substance n2 use*)) or AB ((substance* n2 disorder*) or (drug* n2 addict*) or (abus* n2 alcohol*) or (addict* n2 alcohol*) or (abus* n2 drug*) or (drug* n2 depend*) or (substance* n2 abus*) or (substance n2 use*))
128. TI (sleep* n2 (quality or hygien*) or AB (sleep* n2 (quality or hygien*)
129. TI ((occupational* or physical*) n2 exposure) or AB ((occupational* or physical*) n2 exposure)
130. TI (perform* n3 (adjust* or manip*) or AB (perform* n3 (adjust* or manip*)
131. TI (posture* or position*) or AB (posture* or position*)
132. TI (comorbid* OR (chronic* adj2 disease*) OR (heart* adj2 disease*) or stroke* or cancer* or tumour* or tumor* or neoplas* or metast* or malignan* OR diabet*)) or AB (comorbid* OR (chronic* adj2 disease*) OR (heart* adj2 disease*) or stroke* or cancer* or tumour* or tumor* or neoplas* or metast* or malignan* OR diabet*))

133.OR/103-132 [* Risk Factors]**

134. MH Survey
135. MH Structured Questionnaires
136. MH Cross Sectional Studies
137. MH Epidemiological Research
138. MH Prospective Studies +
139. TI (survey* or questionnaire*) OR AB (survey* or questionnaire*)
140. TI cohort* or AB cohort*
141. TI follow-up* or followup* or AB follow-up* or followup* or TI followed up or AB followed up or TI followed-up or AB followed-up
142. TI longitudinal* or AB longitudinal*
143. TI prospective* or AB prospective* or TI retrospective* or AB retrospective* or TI comparative n2 (study or studies or analys*) or AB comparative* n2 (study or studies or analys*) or TI observational n2 (study or studies or analys*) or AB observational* n2 (study or studies or analys*)
144. comparative stud* or (cohort and (study or studies or analys*)) or (case adj control*) or (comparative and (study or studies))
145. PT observational

146. TI cross-section* OR AB cross-section* or TI (cross* n2 section*) or Ab (cross* n2 section*)

147. OR /134-147 [** Study Design]

148. 78 and 91 and (102 or 133 or 147)

149. 148 NOT PT (comment or clinical conference or congress or consensus development conference or editorial or letter or review or systematic review or guideline or practice guideline or case reports)

Database & Platform: EMBASE (Ovid)

Investigator: Dr Lauren Ead

Librarian: Kent Murnaghan

Years of search: Inception - current **Languages**: No limit

Date search run: MAY 1 2023 **Number of records retrieved: 1010**

Search Strategy:

1. Low Back Pain/
2. Exp Backache/
3. Intervertebral Disk Hernia/
4. Lumbar Disk Hernia/
5. Intervertebral Disk/
6. Piriformis Syndrome/
7. Spondylosis/
8. Radiculopathy/
9. Sciatica/
10. Exp Spine Disease/
11. Vertebral Canal Stenosis/
12. Lumbar Spinal Stenosis/
13. Spine Injury/
14. Spondylolysis/
15. Osteoarthritis/
16. Synovial Cyst/
17. Occupational Accident/
18. Occupational Disease/
19. Musculoskeletal System/
20. Musculoskeletal Disease/
21. Neuromuscular Disease/
22. (low* adj2 (back adj2 (pain* or facet or (nerve adj2 root*) or osteoarth* or radicul* or stenos* or spondylo* or injur* or discomfort* or dysfunction* or sore* or herniat* or trauma* or sprain* or strain* or ach*))).mp.

23. (low* adj2 (trunk* adj2 (pain* or facet or (nerve adj2 root*) or osteoarth* or radicul* or stenos* or spondylo* or injur* or discomfort* or dysfunction* or sore* or herniat* or trauma* or sprain* or strain* or ach*))).mp.
24. (low* adj2 (back-pain* or back-injur* or trunk-pain*)).mp.
25. (lumbar* adj3 (pain* or facet or (nerve adj2 root*) or osteoarth* or radicul* or stenos* or spondylo* or zygapophys* or injur* or discomfort or dysfunction* or sore* or herniat* or trauma* or sprain* or strain* or ach*)).mp.
26. lumbo* adj3 (pain* or facet or (nerve adj2 root*) or osteoarth* or radicul* or stenos* or spondylo* or zygapophys* or injur* or discomfort or dysfunction* or sore* or herniat* or trauma* or sprain* or astrain* or ach*).mp.
27. (lumbar* adj3 (disc* adj3 (extru* or degenerat* or displac* or herniat* or prolaps* or sequestered* or slipped* or protru* or avuls*))).mp.
28. (lumbar* adj3 (disk* adj3 (extru* or degenerat* or displac* or herniat* or prolaps* or sequestered* or slipped* or protru* or avuls*))).mp.
29. (back adj2 (pain* or facet or (nerve adj2 root*) or osteoarth* or radicul* or stenos* or spondylo* or injur* or discomfort* or dysfunction* or sore* or herniat* or trauma* or sprain* or strain* or ach*))).mp.
30. back-pain*.mp.
31. (backach* or back-ach*).mp.
32. (back adj2 muscle*) adj2 pain*.mp.
33. (intervertebral* adj3 (disc* adj3 (extru* or degenerat* or displac* or herniat* or prolaps* or sequestered or slipped or protru* or avuls*))) .mp.
34. (intervertebral* adj3 (disk* adj3 (extru* or degenerat* or displac* or herniat* or prolaps* or sequestered or slipped or protru* or avuls*))) .mp.
35. (vertebral* adj3 (disc* adj3 (extru* or degenerat* or displac* or herniat* or prolaps* or sequestered or slipped or protru* or avuls*))) .mp.
36. (vertebral* adj3 (disk* adj3 (extru* or degenerat* or displac* or herniat* or prolaps* or sequestered or slipped or protru* or avuls*))) .mp.
37. coccydyn*.mp.
38. (coccyx* adj2 (pain* or facet or (nerve adj2 root*) or osteoarth* or radicul* or stenos* or spondylo* or zygapophys* or injur* or discomfort* or dysfunction* or sore* or herniat* or sprain* or strain* or trauma*)).mp.
39. (coccygodyn* or coccalg*).mp.
40. coccygalg*.mp.
41. coccygeal* adj2 (pain* or facet or (nerve adj2 root*) or osteoarth* or radicul* or stenos* or spondylo* or zygapophys* or injur* or discomfort* or dysfunction* or sore* or herniat* or sprain* or strain* or trauma*).mp.
42. dorsalg*.mp.
43. lumbago*.mp.
44. lumboischialg*.mp.
45. piriformis* adj2 syndrome*.mp.
46. sacral* adj3 (pain* or facet or nerve adj2 root* or osteoarth* or radicul* or stenos* or spondylo* or zygapophys* or injur* or discomfort or dysfunction* or sore* or herniat* or sprain* or strain* or trauma* or ach*).mp.

47. sacro* adj3 (pain* or facet or nerve adj2 root* or osteoarth* or radicul* or stenos* or spondylo* or zygapophys* or injur* or discomfort or dysfunction* or sore* or herniat* or sprain* or strain* or trauma* or ach*).mp.
48. sacrum* adj2 (pain* or facet or (nerve adj2 root*) or osteoarth* or radicul* or stenos* or spondylo* or zygapophys* or injur* or discomfort or dysfunction* or sore* or herniat* or sprain* or strain* or trauma* or ach*).mp.
49. SI adj2 (joint* adj2 (pain* or facet or (nerve adj2 root*) or osteoarth* or radicul* or stenos* or spondylo* or zygapophys* or injur* or discomfort or dysfunction* or sore* or herniat* or sprain* or strain* or ach*)).mp.
50. sciatic*.mp.
51. stenos* adj2 (spine* or spinal* or vertebral*).mp.
52. (spine or spinal) adj2 osteoarthr*.mp.
53. spine* adj3 (condition* or diseas* or disab* or disorder* or degen* or pain* or stenos*).mp.
54. spinal* adj3 (condition* or diseas* or disab* or disorder* or degen* or pain* or stenos*).mp.
55. spondylosis*.mp.
56. tailbone* adj3 (pain* or facet or (nerve adj2 root*) or osteoarth* or radicul* or stenos* or spondylo* or injur* or discomfort* or dysfunction* or sore* or herniat* or sprain* or strain* or ach*)).mp.
57. vertebr* adj3 (pain* or facet or (nerve adj2 root*) or osteoarth* or radicul* or stenos* or spondylo* or injur* or discomfort* or dysfunction* or sore* or herniat* or sprain* or strain* or ach*)).mp
58. (poly-radicul* or polyradicul*).mp.
59. neuropath* adj2 (lumbar* or lumbo* or sacral* or sacro* or (low* adj2 back) or low-back* or lower-back* or spine* or spinal* or L1 or L2 or L3 or L4 or L5).mp.
60. radiculopath* adj3 (lumbar* or lumbo* or sacral* or sacro* or (low* adj2 back) or low-back* or lower-back* or spine* or spinal* or L1 or L2 or L3 or L4 or L5).mp.
61. radiating* adj3 (lumbar* or lumbo* or sacral* or sacro* or (low* adj2 back) or low-back* or lower-back* or spine* or spinal* or L1 or L2 or L3 or L4 or L5).mp.
62. radicular* adj3 (lumbar* or lumbo* or sacral* or sacro* or (low* adj2 back) or low-back* or lower-back* or spine* or spinal* or L1 or L2 or L3 or L4 or L5).mp.
63. (lumborum* or longissimus* or (erector* adj2 spin*)).mp.
64. synovial* adj2 cyst*.mp.
65. thoracolumbar* adj2 (pain* or facet* or nerve* adj2 root* or osteoarthr* or radicul* or stenos* or spondylo* or injur* or trauma* or discomfort* or dysfunction* or sore* or herniat* or sprain* or strain* or trauma* or ach*).mp.
66. thoraco-lumbar* adj2 (pain* or facet* or nerve* adj2 root* or osteoarthr* or radicul* or stenos* or spondylo* or zygapohys* or injur* or trauma* or discomfort* or dysfunction* or sore* or herniat* or sprain* or strain* or trauma* or ach*).mp.
67. curvatur* adj2 (spine* or spinal*).mp.
68. (pathol* adj2 (lumbar* or (low* adj2 back) or low-back* or (lower* adj2 back) or lower-back* or thoracolumbar* or thoraco-lumbar* or intervertebral* or lumbosacral* or lumbo-sacral* or sacral* or sacro-iliac* or sacroiliac*)).mp.
69. (disease* or injur* or disorder* or complaint*) adj2 (work* or occupation* or musculoskeletal* or neuromuscul* or overuse*).mp.

70. repetitive* adj2 (sprain* or strain*).mp.
71. OR /1-70 [* Low Back Pain]**

72. Exp Student/
73. (student* or trainee* or intern or interns or residen*) .mp.
74. Chiropractic/
75. chiropr*.mp.
76. chiropractor*.mp.
77. (doctor* or physician* or practitioner* or clinician* or practice* or provider*).mp.
78. (doctor* or physician* or practitioner* or clinician* or practice* or provider*) adj2 chiropr*).mp.
79. or/72-73
80. or/74-75
81. 79 AND 80
82. 74 AND 77
83. OR/ 76 or 78 or 81 or 82 [* Chiropractors/ Chiropractic Students]**

84. Incidence/
85. Prevalence/
86. incidence*.mp.
87. prevalen*.mp.
88. occurrence* .mp.
89. rate*.mp.
90. proportion*.mp.
91. first onset.mp.
92. first episode.mp.
93. OR /84-92 [* Incidence/ Prevalence]**

94. Risk Factor/
95. Sociodemographics/
96. Social Environment/
97. Mental Health/
98. Mental Stress/
99. exp Comorbidity/
100. exp Chronic Disease/
101. exp Heart Disease/
102. exp Cerebrovascular Accident/
103. exp Neoplasm/
104. exp Diabetes Mellitus/
105. exp Exercise/
106. Sedentary Lifestyle/
107. Workplace/
108. Substance Abuse /
109. Sleep Quality/
110. Sleep Disorder/

111. Occupational Exposure/
112. Body Position/
113. ((risk* adj2 factor*) or (associated* adj2 factor*)).mp.
114. (comorbid* OR (chronic* adj2 disease*) OR (heart* adj2 disease*) or stroke* or cancer* or tumour* or tumor* or neoplas* or metast* or malignan* OR diabet*).mp.
115. health* adj2 correlat*.mp.
116. ((sociodemographic* adj2 factor*) or (socio-demographic* adj2 factor*) or sex or gender* or income or (employment* adj2 status*) or (social* adj2 environment*)) .mp.
117. ((psychological* adj2 distress*) or (mental* adj2 distress*) or stress* or anxiety* or anxious* or depress* or (mental* adj2 health*) or psychosocial* or psycho-social*).mp.
118. ((physical* adj2 activit*) or (resistance* adj2 training*) or (resistance* adj2 activit*) or (aerobic* adj2 training*) or (aerobic* adj2 activit*) or (endurance* adj2 training*) or (endurance* adj2 activit*) or (strength* adj2 training*) or (strength* adj2 activit*) or (activit* adj2 level*) or exercis*).mp.
119. ((sedentary* adj2 behavior*) or (sedentary* adj2 behaviour*) or (physical* adj2 inactivit*)).mp.
120. ((substance-related* adj2 disorder*) or (drug* adj2 addict*) or (alcohol* adj2 abus*) or (alcohol* adj2 addict*) or (drug* adj2 abus*) or (drug* adj2 depend*) or substance-use* or (substance* adj2 use*)).mp.
121. ((sleep* adj2 qualit*) or (sleep* adj2 hygien*)).mp.
122. ((occupational* adj2 exposur*) or (physical* adj2 exposur*) or postur* or (perform* adj3 (manip* or adjust*)) or position*) .mp.

123. OR /94-122 [**Risk Factors]

124. Exp Questionnaire/
125. Cross-Sectional Study/
126. Cohort Analysis/
127. Case-Control Study/
128.Observational Study/
129. Longitudinal Study/
130. Retrospective Study/
131. Prospective Study/
132. Epidemiology/
133. (survey* or questionnair* or cross-section* or (cross* adj1 section*) or cohort* or (observational* adj2 (study or studies or analy*)) or follow-up* or prospective* or retrospectiv* or longitudinal*) .mp.

134. **OR /124-133 [** Study Design]**

135. 71 and 83 and (93 or 123 or 134)
[LBP and Chiropractors/Chiro Students and (Prevalence OR Risk Factors OR Study Design)]

136. 135 NOT (books or chapter or conference abstract or conference paper or conference review or review or editorial or letter or review).pt.

Database & Platform: PsycINFO (Ovid)

Investigator: Dr Lauren Ead

Librarian: Kent Murnaghan

Years of search: Inception - current **Languages**: No limit

Date search run: MAY 1 2023 **Number of records retrieved:** **55**

Search Strategy:

1. exp Back Pain/
2. (low* adj2 (back adj2 (pain* or facet or (nerve adj2 root*) or osteoarth* or radicul* or stenos* or spondylo* or injur* or discomfort* or dysfunction* or sore* or herniat* or trauma* or sprain* or strain* or ach*))).mp.
3. (low* adj2 (trunk* adj2 (pain* or facet or (nerve adj2 root*) or osteoarth* or radicul* or stenos* or spondylo* or injur* or discomfort* or dysfunction* or sore* or herniat* or trauma* or sprain* or strain* or ach*))).mp.
4. (low* adj2 (back-pain* or back-injur* or trunk-pain*)).mp.
5. (lumbar* adj3 (pain* or facet or (nerve adj2 root*) or osteoarth* or radicul* or stenos* or spondylo* or zygapophys* or injur* or discomfort or dysfunction* or sore* or herniat* or trauma* or sprain* or strain* or ach*)).mp.
6. lumbo* adj3 (pain* or facet or (nerve adj2 root*) or osteoarth* or radicul* or stenos* or spondylo* or zygapophys* or injur* or discomfort or dysfunction* or sore* or herniat* or trauma* or sprain* or astrain* or ach*).mp.
7. (lumbar* adj3 (disc* adj3 (extru* or degenerat* or displac* or herniat* or prolaps* or sequestered* or slipped* or protru* or avuls*))).mp.
8. (lumbar* adj3 (disk* adj3 (extru* or degenerat* or displac* or herniat* or prolaps* or sequestered* or slipped* or protru* or avuls*))).mp.
9. (back adj2 (pain* or facet or (nerve adj2 root*) or osteoarth* or radicul* or stenos* or spondylo* or injur* or discomfort* or dysfunction* or sore* or herniat* or trauma* or sprain* or strain* or ach*))).mp.
10. back-pain*.mp.
11. (backach* or back-ach*).mp.
12. (back adj2 muscle*) adj2 pain*.mp.
13. (intervertebral* adj3 (disc* adj3 (extru* or degenerat* or displac* or herniat* or prolaps* or sequestered or slipped or protru* or avuls*))) .mp.
14. (intervertebral* adj3 (disk* adj3 (extru* or degenerat* or displac* or herniat* or prolaps* or sequestered or slipped or protru* or avuls*))) .mp.
15. (vertebral* adj3 (disc* adj3 (extru* or degenerat* or displac* or herniat* or prolaps* or sequestered or slipped or protru* or avuls*))) .mp.
16. (vertebral* adj3 (disk* adj3 (extru* or degenerat* or displac* or herniat* or prolaps* or sequestered or slipped or protru* or avuls*))) .mp.
17. coccydyn*.mp.
18. (coccyx* adj2 (pain* or facet or (nerve adj2 root*) or osteoarth* or radicul* or stenos* or spondylo* or zygapophys* or injur* or discomfort* or dysfunction* or sore* or herniat* or sprain* or strain* or trauma*)).mp.
19. (coccygodyn* or coccalg*).mp.
20. coccygalg*.mp.

21. coccygeal* adj2 (pain* or facet or (nerve adj2 root*) or osteoarth* or radicul* or stenos* or spondylo* or zygapophys* or injur* or discomfort* or dysfunction* or sore* or herniat* or sprain* or strain* or trauma*).mp.
22. dorsalg*.mp.
23. lumbago*.mp.
24. lumboischialg*.mp.
25. piriformis* adj2 syndrome*.mp.
26. sacral* adj3 (pain* or facet or nerve adj2 root* or osteoarth* or radicul* or stenos* or spondylo* or zygapophys* or injur* or discomfort or dysfunction* or sore* or herniat* or sprain* or strain* or trauma* or ach*).mp.
27. sacro* adj3 (pain* or facet or nerve adj2 root* or osteoarth* or radicul* or stenos* or spondylo* or zygapophys* or injur* or discomfort or dysfunction* or sore* or herniat* or sprain* or strain* or trauma* or ach*).mp.
28. sacrum* adj2 (pain* or facet or (nerve adj2 root*) or osteoarth* or radicul* or stenos* or spondylo* or zygapophys* or injur* or discomfort or dysfunction* or sore* or herniat* or sprain* or strain* or trauma* or ach*).mp.
29. SI adj2 (joint* adj2 (pain* or facet or (nerve adj2 root*) or osteoarth* or radicul* or stenos* or spondylo* or zygapophys* or injur* or discomfort or dysfunction* or sore* or herniat* or sprain* or strain* or ach*)).mp.
30. sciatic*.mp.
31. stenos* adj2 (spine* or spinal* or vertebral*).mp.
32. (spine or spinal) adj2 osteoarthr*.mp.
33. spine* adj3 (condition* or diseas* or disab* or disorder* or degen* or pain* or stenos*).mp.
34. spinal* adj3 (condition* or diseas* or disab* or disorder* or degen* or pain* or stenos*).mp.
35. spondylosis*.mp.
36. tailbone* adj3 (pain* or facet or (nerve adj2 root*) or osteoarth* or radicul* or stenos* or spondylo* or injur* or discomfort* or dysfunction* or sore* or herniat* or sprain* or strain* or ach*)).mp.
37. vertebr* adj3 (pain* or facet or (nerve adj2 root*) or osteoarth* or radicul* or stenos* or spondylo* or injur* or discomfort* or dysfunction* or sore* or herniat* or sprain* or strain* or ach*)).mp
38. (poly-radicul* or polyradicul*).mp.
39. neuropath* adj2 (lumbar* or lumbo* or sacral* or sacro* or (low* adj2 back) or low-back* or lower-back* or spine* or spinal* or L1 or L2 or L3 or L4 or L5).mp.
40. radiculopath* adj3 (lumbar* or lumbo* or sacral* or sacro* or (low* adj2 back) or low-back* or lower-back* or spine* or spinal* or L1 or L2 or L3 or L4 or L5).mp.
41. radiating* adj3 (lumbar* or lumbo* or sacral* or sacro* or (low* adj2 back) or low-back* or lower-back* or spine* or spinal* or L1 or L2 or L3 or L4 or L5).mp.
42. radicular* adj3 (lumbar* or lumbo* or sacral* or sacro* or (low* adj2 back) or low-back* or lower-back* or spine* or spinal* or L1 or L2 or L3 or L4 or L5).mp.
43. (lumborum* or longissimus* or (erector* adj2 spin*)).mp.
44. synovial* adj2 cyst*.mp.

45. thoracolumbar* adj2 (pain* or facet* or nerve* adj2 root* or osteoarthr* or radicul* or stenos* or spondylo* or injur* or trauma* or discomfort* or dysfunction* or sore* or herniat* or sprain* or strain* or trauma* or ach*).mp.
46. thoraco-lumbar* adj2 (pain* or facet* or nerve* adj2 root* or osteoarthr* or radicul* or stenos* or spondylo* or zygapohys* or injur* or trauma* or discomfort* or dysfunction* or sore* or herniat* or sprain* or strain* or trauma* or ach*).mp.
47. curvatur* adj2 (spine* or spinal*).mp.
48. (pathol* adj2 (lumbar* or (low* adj2 back) or low-back* or (lower* adj2 back) or lower-back* or thoracolumbar* or thoraco-lumbar* or intervertebral* or lumbosacral* or lumbo-sacral* or sacral* or sacro-iliac* or sacroiliac*)).mp.
49. (disease* or injur* or disorder* or complaint*) adj2 (work* or occupation* or musculoskeletal* or neuromuscul* or overuse*).mp.
50. repetitive* adj2 (sprain* or strain*).mp.

51. OR /1-50 [* Low Back Pain]**

52. Exp Students/
53. (student* or trainee* or intern or interns or residen*) .mp.
54. Chiropractic/
55. chiropr*.mp.
56. chiropractor*.mp.
57. (doctor* or physician* or practitioner* or clinician* or practice* or provider*).mp.
58. (doctor* or physician* or practitioner* or clinician* or practice* or provider*) adj2 chiropr*).mp.
59. or/52-53
60. or/54-55
61. 59 AND 60
62. 54 AND 57

63. OR/ 56 or 58 or 61 or 62 [* Chiropractors/ Chiropractic Students]**

64. incidence*.mp.
65. prevalen*.mp.
66. occurrence* .mp.
67. rate*.mp.
68. proportion*.mp.
69. first onset.mp.
70. first episode.mp.

71. OR /64-70 [* Incidence/ Prevalence]**

72. Risk Factors/
73. Sociodemographic Factors/
74. Demographic Characterisitics/
75. Exp Social Environments/
76. Exp Mental Health/
77. Exp Stress, Psychological/
78. exp Comorbidity/

79. exp Chronic Illness/
80. exp Cardiovascular Disorders/
81. Exp Heart Disorders/
82. Exp Cerebrovascular Accidents/
83. exp Neoplasms /
84. exp Diabetes Mellitus/
85. exp Exercise/
86. Exp Sedentary Behavior/
87. Exp Substance-Use Disorder/
88. Exp Substance-related and Addictive Disorders/
89. Exp Sleep Quality/
90. Exp Sleep Deprivation/
91. Exp Occupational Exposure/
92. Exp Posture/
93. ((risk* adj2 factor*) or (associated* adj2 factor*)).mp.
94. (comorbid* OR (chronic* adj2 disease*) OR (heart* adj2 disease*) or stroke* or cancer* or tumour* or tumor* or neoplas* or metast* or malignan* OR diabet*).mp.
95. health* adj2 correlat*.mp.
96. ((sociodemographic* adj2 factor*) or (socio-demographic* adj2 factor*) or sex or gender* or income or (employment* adj2 status*) or (social* adj2 environment*)) .mp.
97. ((psychological* adj2 distress*) or (mental* adj2 distress*) or stress* or anxiety* or anxious* or depress* or (mental* adj2 health*) or psychosocial* or psycho-social*).mp.
98. ((physical* adj2 activit*) or (resistance* adj2 training*) or (resistance* adj2 activit*) or (aerobic* adj2 training*) or (aerobic* adj2 activit*) or (endurance* adj2 training*) or (endurance* adj2 activit*) or (strength* adj2 training*) or (strength* adj2 activit*) or (activit* adj2 level*) or exercis*).mp.
99. ((sedentary* adj2 behavior*) or (sedentary* adj2 behaviour*) or (physical* adj2 inactivit*)).mp.
100. ((substance-related* adj2 disorder*) or (drug* adj2 addict*) or (alcohol* adj2 abus*) or (alcohol* adj2 addict*) or (drug* adj2 abus*) or (drug* adj2 depend*) or substance-use* or (substance* adj2 use*)).mp.
101. ((sleep* adj2 qualit*) or (sleep* adj2 hygien*)).mp.
102. ((occupational* adj2 exposur*) or (physical* adj2 exposur*) or postur* or (perform* adj3 (manip* or adjust*)) or position*) .mp.
103. OR /72-102 [**Risk Factors]

104. Exp Surveys/
105. Exp Questionnaires/
106. Cross-Sectional Studies/
107. exp Cohort Analysis/
108. Exp Prospective Studies/
109. Exp Retrospective Studies/
110. (survey* or questionnair* or cross-section* or (cross* adj1 section*) or cohort* or (observational* adj2 (study or studies or analy*)) or follow-up* or prospective* or retrospectiv* or longitudinal*) .mp.
111. **OR /104-110 [** Study Design]**

112. 51 and 63 and (71 or 103 or 111)
[LBP and Chiropractors/Chiro Students and (Prevalence OR Risk Factors OR Study Design)]
